# The prevalence and correlates of vision impairment and glasses ownership among ethnic minority and Han schoolchildren in rural China

**DOI:** 10.1371/journal.pone.0256565

**Published:** 2021-08-30

**Authors:** Huan Wang, Brandon Barket, Sharon Du, Dimitris Friesen, Ezra Kohrman, Esther Tok, Baixiang Xiao, Wenyong Huang, Ving Fai Chan, Graeme MacKenzie, Nathan Congdon

**Affiliations:** 1 Stanford Center on China’s Economy and Institutions, Stanford University, California, Stanford, United States of America; 2 Affiliated Eye Hospital of Nanchang University, Nanchang, China; 3 State Key Laboratory of Ophthalmology and Division of Preventive Ophthalmology, Zhongshan Ophthalmic Center, Sun Yat-sen University, Guangzhou, China; 4 School of Medicine, Dentistry and Biomedical Sciences, Queen’s University Belfast, Belfast, Northern Ireland, United Kingdom; 5 Clearly Initiatives, London, England, United Kingdom; 6 Orbis International, New York, NY, United States of America; Cairo University Kasr Alainy Faculty of Medicine, EGYPT

## Abstract

**Purpose:**

To determine the prevalence of visual impairment and glasses ownership among Han Chinese and Hui minority junior high school children in Ningxia Hui Autonomous Region, China.

**Design:**

Population-based cross-sectional study.

**Methods:**

Vision screening was conducted on 20,376 children (age 12–15 years) in all 124 rural junior high schools in Ningxia. Personal and family characteristics, glasses ownership, and academic performance were assessed through a survey questionnaire and standardized mathematics test, respectively.

**Results:**

The prevalence of visual acuity (VA) ≤6/12 in either eye was significantly higher among Han (54.5%) than Hui (45.2%) children (P<0.001), and was significantly positively associated with age, female sex, Han ethnicity, parental outmigration for work, shorter time spent outside during recess, shorter time spent watching television and higher time spent studying. Among children with VA≤6/12 in both eyes, only 56.8% of Han and 41.5% of Hui children had glasses (P<0.001). Glasses ownership was significantly associated with worse vision, greater family wealth, female sex, higher test scores, age, parental outmigration for work, understanding of myopia and glasses, higher time spent studying and Han ethnicity.

**Conclusion:**

One of the first of its kind, this report on Han and Hui ethnic schoolchildren confirms a high prevalence of visual impairment among both populations, but slightly higher among the Han. Both groups, especially the Hui, have low rates of glasses ownership. Future interventions and policies designed to improve glasses usage should focus on populations with lower incomes and seek to correct erroneous beliefs about the safety of glasses and efficacy of traditional eye exercises.

## Introduction

Visual impairment is among the most common health problems worldwide, comprising half of all disabilities among young people [1]. While most (>90%) cases of visual impairment can be corrected inexpensively using prescription glasses, rates of glasses ownership remain low in underserved populations, in some areas meeting only 15% of existing need [2–9].

Strong evidence from multiple trials suggests that visual impairment impedes children’s education in measurable ways [8, 10–12]. Children with visual impairment also have lower scores in a variety of motor, cognitive, and reading tests [13–15]. Correcting vision with glasses has been shown to significantly increase children’s learning outcomes [8, 10, 11].

As many as half of children suffering from visual impairment due to uncorrected refractive error globally live in China [16]. Among these children, myopia is responsible for over 90% of visual impairment [17, 18]. Meanwhile, ownership of glasses among children needing them remains as low as 15–20% among rural and urban migrant children in China [8, 9], despite recent attention on the part of the government to addressing the issue of childhood myopia with a national program [19].

Existing research on visual impairment in China focuses almost exclusively on persons of Chinese Han descent [8, 17, 20]. Rates of visual impairment due to refractive error in rural Han children increase from 24% in primary school to 54% in high school [17, 18, 21], while for urban Han children, prevalence of visual impairment reaches as high as 80% in high school [20]. Yet, despite the increasing availability of research on Han children, little is known about visual impairment and glasses ownership among China’s ethnic minorities, who number over 100 million people [22]. Based on the limited available data, prevalence of vision impairment such as refractive error appears to vary widely between Han Chinese and their minority counterparts. For example, one study found members of the Yi minority in Yunnan had only half the refractive error prevalence of their Han neighbors (16.8% vs 31.5%) [23]. However, few studies have examined the prevalence of vision impairment and glasses ownership among older cohorts of minority children, particularly in China’s poor northwest [24, 25].

There is almost no published evidence regarding visual impairment and glasses ownership among the Hui population, the second-largest minority group in China, numbering over 10 million people [22]. Existing studies report small, non-randomly-selected populations and provide limited information on lifestyle factors known to impact visual impairment risk [26]. Prior literature has not considered the socioeconomic disparities between Han and Hui populations: the Hui reside predominantly in rural areas [27], and rural Hui per capita income is about 20% less than their Han counterparts [28].

The objective of the current study is to determine the prevalence and personal, family and lifestyle-related predictors of visual impairment and glasses ownership in a majority-Hui community in China’s predominantly rural northwestern Ningxia Hui Autonomous Region to better guide interventions and government policies aiming to improve eye health in underserved areas.

## Methods

The protocol for this study was approved by the Institutional Review Boards at Stanford University (Palo Alto, USA), Queen’s University Belfast (Belfast, Northern Ireland), and the Zhongshan Ophthalmic Center, Sun Yat-sen University (Guangzhou, China). Permission was received from the local Board of Education and the principals of all junior high schools in Ningxia. Written informed consent was provided from at least one parent for each child participant. The principles of the Declaration of Helsinki were followed throughout.

### Setting

Ningxia is a landlocked autonomous region located in the northwest of China, with a total area of 66,400 square kilometers. The province has a population of approximately 6.62 million people, of which 62% are of Han ethnicity and 38% are Hui, and is largely composed of sparsely-settled desert, relying on an extensive system of canals and irrigation to support agricultural production. In 2018, Ningxia’s per capita gross domestic product was $8,174, about 20% lower than the national figure of $9,769 in the same year [22, 29].

The Hui ethnic minority consists of Chinese-speaking practitioners of Islam, a religion historically popularized by Silk Road traders from Central Asia and the Middle East, who intermarried with the local population. The Hui people are predominantly concentrated in northwestern provinces such as Ningxia, Gansu, Qinghai and Xinjiang. Genetic evidence suggests Hui people have a high degree of homogeneity with co-resident Han Chinese populations [30], though Hui participants demonstrated 3.66% European-related ancestry in one study [31].

### Sampling and eligibility criteria

The data analyzed in this study were collected during a survey and visual acuity (VA) assessment conducted as part of a randomized trial in October of 2019. To obtain a representative sample of rural junior high children in Ningxia, we followed a two-step selection protocol. We first obtained a list of all 273 junior high schools from local education bureaus in all 6 prefectures in Ningxia. To focus on rural school children, we excluded 122 schools located in the capital city of Yinchuan, urban centers of prefectures and county seats. We also excluded schools with fewer than 40 children in the 7^th^ grade (18% of the sample frame) due to logistical constraints. After these exclusion criteria, 124 junior high schools were included in our sample.

The second step involved selecting sample classes and children in the 7^th^ and 8^th^ grades (age range 12–15 years). We randomly selected at most two classes from each grade in each school, and surveyed every child in the sample classes. In total, 20,375 children in 474 classes in 124 sample schools were enrolled.

### Questionnaires

Enumerators administered questionnaires to the sampled children, collecting information on age, sex, parental education level and parental migration status, self-reported outdoor activity during recess, belief whether glasses can harm vision, belief that eye exercises can prevent or correct myopia, family wealth, self-reported time spent watching television after school, self-reported time spent studying after school, and glasses ownership. Each child was also administered a 35-minute standardized mathematics test by study personnel.

### Visual acuity assessment

Children underwent VA screening at each school by an optometrist and trained research assistant (graduate students from local universities). VA was tested separately for each eye without refraction at 4 m using Early Treatment Diabetic Retinopathy tumbling E study charts (Precision Vision, La Salle, IL) [8] in a well-lit, indoor area. We have expressed VA results in Snellen fraction instead of logMAR values, as this is more familiar to the majority of readers. If children correctly identified the orientation of at least four of five optotypes on the 6/60 line, they were examined on the 6/30 line, then the 6/15 line, and then line by line to 6/3. We defined VA for an eye as the lowest line on which four of five optotypes were read correctly. If the 6/60 line could not be read at 4 m, the participant was tested at 1 m and the measured VA was divided by 4.

### Statistical analysis

Following the Refractive Error Studies in Children (RESC) protocol, we defined visual impairment as uncorrected VA ≤6/12 in either eye [32]. Glasses ownership was defined as the ability to produce glasses at school at the time of the baseline survey, among children with VA≤6/12 in both (rather than either) eyes. We used this stricter cutoff to capture glasses ownership among those children who had a need for correction in both eyes. Standardized math score was calculated for each grade separately to give a mean of 0 and SD of 1. We calculated family wealth by summing the value of self-reported possessions from a pre-defined list, as reported in the China Rural Household Survey Yearbook [33].

Relative risk (RR) estimation using a generalized linear model with a Poisson regression and robust error adjustment for clustering by school was used to determine the association between outcomes of interest (visual impairment and glasses ownership) and child characteristics, including ethnicity, age, sex, parental education, parental migration, self-reported time allocation (time spent on outdoor activities, watching television and studying), standardized mathematics test score, family wealth, belief that wearing glasses harms vision and belief that eye exercises can correct myopia.

A multiple linear regression model was used to conduct the multivariate analysis. All variables that were statistically significant (p<0.05) in the univariate analysis were included in the multivariate regression model.

All analyses were performed using Stata 16.0 (Stata Corp, College Station, TX). P-values < 0.05 were considered statistically significant. Relative risk (RR) and 95% confidence interval (CI) were presented. Robust standard errors were calculated, adjusting for clustering by school.

To reduce the inefficiency of estimation due to missing values, multiple imputation in Stata was used to impute data for several variables, including belief that wearing glasses harms vision (n = 3), parental education level (n = 5), parental migration status (n = 5), and time allocated for outdoor activity (n = 2).

## Results

Among the Hui children in our sample, 49.3% were girls, a larger proportion than that among Han children (45.4%, P<0.001) (Table 1). A total of 42.9% Hui children thought that glasses worsened vision, significantly larger than the 40.5% among Han children (P = 0.001). Among Hui children, 58.4% thought that eye exercises could correct or prevent visual impairment such as myopia, larger than the 53.6% of Han (P<0.001) (Table 1).

**Table 1:** Demographic, economic and attitudinal factors potentially predicting visual impairment and glasses ownership, stratified by ethnicity.

	All (n = 20376)	Han (n = 7140, 35.1%)	Hui (n = 13236, 64.9%)	P-value comparing Hui and Han students
Age, years, mean (SD)	13.5 (1.1)	13.4 (0.9)	13.5 (1.2)	<0.001
Female sex	9762 (47.9)	3238 (45.4)	6524 (49.3)	<0.001
One or both parents with ≥9 years of education	11314 (55.5)	5456 (76.4)	5858 (44.3)	<0.001
One or both parents out-migrated for work	7285 (35.8)	2624 (36.8)	4661 (35.2)	0.029
Any self-reported outdoor activity during recess	6991 (34.3)	2347 (32.8)	4644 (35.1)	0.001
Think eyeglasses will harm vision	8575 (42.1)	2890 (40.5)	5685 (42.9)	0.001
Think eye exercises can correct myopia	11559 (56.7)	3827 (53.6)	7732 (58.4)	<0.001
Standardized math score (bottom third)	7052 (34.6)	2002 (28.0)	5050 (38.2)	<0.001
Family wealth (poorest third)	6828 (33.5)	1958 (27.4)	4870 (36.8)	<0.001
No time spent watching television after school	9755 (47.9)	3698 (51.8)	6057 (45.8)	<0.001
Time spent on studying after school >60 mins	6953 (34.1)	3047 (42.7)	3906 (29.5)	<0.001
Severe vision impairment (VA≤6/60 in better seeing eye)	620 (3.0)	292 (4.1)	328 (2.5)	<0.001

Values are numbers (percentages) unless stated otherwise.

While for many variables, the Hui and Han children were statistically significantly different, these differences were still relatively small. For example, a total of 35.1% of Hui children reported outdoor activity during recess, while 32.8% of Han did the same (P = 0.001). Among Hui (35.2%) and Han (36.8%) families, one or both parents out-migrated for work at slightly different rates (P = 0.029) (Table 1).

Hui families tended to be less educated than Han families, as only 44.3% of Hui children had one or both parents with ≥9 years of education, while 76.4% of Han children had the same (P<0.001). Among Hui families, 36.8% were in the lowest tercile of family wealth for the entire sample, far higher than the 27.4% figure for Han (P<0.001). Hui (36.8%) standardized math scores were more likely to be in the bottom tercile than those of the Han (28.0%, P<0.001). Only 29.5% of Hui children spent more than an hour studying after school, while 42.7% of Han reported this level of study (P<0.001) (Table 1).

**Fig 1:**
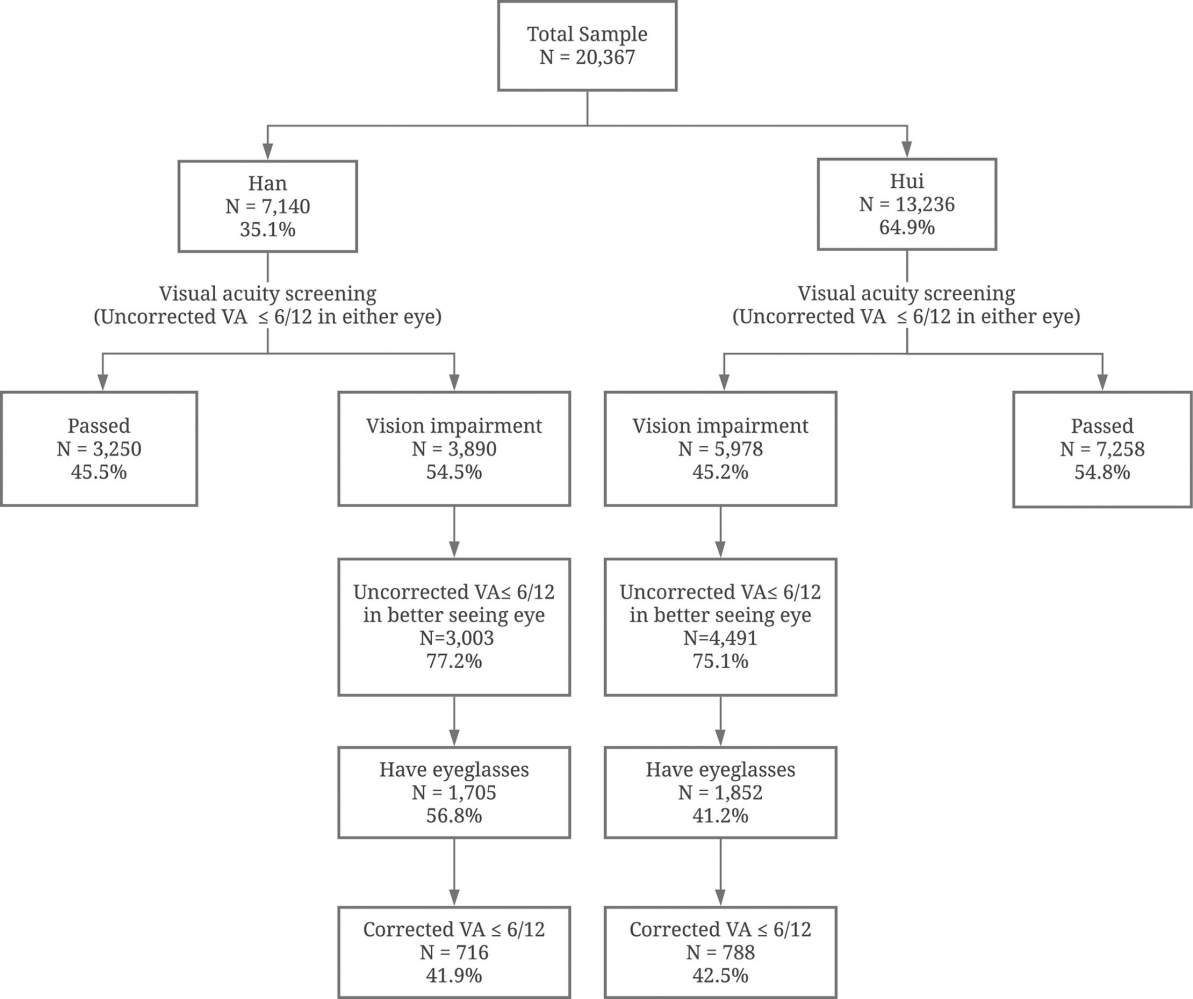
Sample distribution.

Among Hui children, 45.2% suffered from visual impairment (uncorrected VA ≤6/12 in either eye), lower than the Han rate (54.5%, P<0.001) (Fig 1). Among children visually impaired in both eyes, only 41.2% of Hui and 56.8% of Han children had glasses (P<0.001). Among children owning glasses, 42.5% of Hui children and 41.9% of Han children had corrected VA ≤6/12 in either eye, a difference significant at the 1% level (P<0.001, Fig 1).

In the simple regression model (Table 2, Panel A), visual impairment was positively associated with the following: age (RR, 1.04; P<0.001), female sex (RR, 1.42; P<0.001), greater parental education (RR, 1.08; P<0.001), parental out-migration (RR, 1.04; P = 0.026), no time spent watching television after school (RR, 1.10; P<0.001) and spending at least one hour studying after school (RR, 1.14; P<0.001). Visual impairment was negatively associated with self-reported outdoor activity during recess (RR, 0.87; P<0.001), bottom tercile standardized math score (RR, 0.91; P<0.001) and being Hui (RR, 0.83; P<0.001). Table 2 Panel B shows the multivariate model results, visual impairment was associated with age, female sex, parental out-migration, lack of TV watching, after-school study, and self-reported outdoor time persisted, though was not associated with math score and parental education. Being in the lowest tercile of family wealth was not significantly associated with visual impairment.

**Table 2:** Potential risk factors for visual impairment (uncorrected visual acuity < = 6/12 in either eye).

	Total			Han			Hui		
	RR	95% CI	P-value	RR	95% CI	P-value	RR	95% CI	P-value
*Panel A Simple univariate model*									
Age, years	1.04	1.02,1.06	<0.001	1.04	1.01,1.06	0.006	1.05	1.03,1.07	<0.001
Female sex	1.42	1.37,1.47	<0.001	1.35	1.29,1.41	<0.001	1.49	1.43,1.55	<0.001
One or both parents with ≥9 years of education	1.08	1.04,1.13	<0.001	1.04	0.99,1.09	0.092	1.02	0.97,1.07	0.409
One or both parents out-migrated for work	1.04	1.00,1.07	0.026	1.05	0.99,1.10	0.056	1.03	0.99,1.07	0.217
Self-reported outdoor activity during recess, hours	0.87	0.84,0.90	<0.001	0.87	0.83,0.93	<0.001	0.88	0.84,0.91	<0.001
Standardized math score (bottom third)	0.91	0.87,0.95	<0.001	0.94	0.87,1.00	0.057	0.92	0.88,0.97	0.002
Family wealth (poorest third)	0.97	0.93,1.01	0.145	0.98	0.93,1.03	0.434	0.99	0.94,1.04	0.002
No time spent watching television after school	1.10	1.07,1.14	<0.001	1.08	1.03,1.13	0.003	1.10	1.06,1.14	<0.001
Time spent on studying after school>60 mins	1.14	1.09,1.18	<0.001	1.11	1.06,1.16	<0.001	1.11	1.05,1.17	<0.001
Hui student	0.83	0.78,0.88	<0.001	-	-	-	-	-	-
*Panel B Multiple variate model*									
Age, years	1.05	1.04,1.07	<0.001	1.04	1.02,1.07	0.002	1.06	1.04,1.07	<0.001
Female sex	1.41	1.36,1.45	<0.001	1.32	1.26,1.39	<0.001	1.47	1.41,1.53	<0.001
One or both parents with ≥9 years of education	1.03	0.99,1.07	0.110	1.03	0.98,1.08	0.224	1.03	0.98,1.08	0.187
One or both parents out-migrated for work	1.04	1.01,1.07	0.013	1.05	1.00,1.10	0.031	1.03	0.99,1.07	0.178
Self-reported outdoor activity during recess, hours	0.95	0.91,0.98	<0.001	0.93	0.88,0.98	0.011	0.95	0.91,0.99	0.027
Standardized math score (bottom third)	0.96	0.92,1.00	0.067	0.98	0.92,1.04	0.488	0.95	0.90,1.00	0.059
Family wealth (poorest third)	-	-	-	-	-	-	-	-	-
No time spent watching television after school	1.05	1.02,1.08	0.002	1.04	0.99,1.09	0.107	1.06	1.02,1.10	0.004
Time spent on studying after school>60 mins	1.06	1.03,1.08	<0.001	1.07	1.02,1.11	0.006	1.07	1.01,1.12	0.012
Hui student	0.84	0.79,0.88	<0.001	-	-	-	-	-	-
	20,376			7,140			13,236		

While many potential risk factors were correlated with visual impairment for both the Han and the Hui, some were only significant for one group or the other, but not both. In the simple regression model (Table 2, Panel A), parental education (RR, 1.04; P = 0.092) and parental migration (RR, 1.05; P = 0.056) were only significant for the Han, while bottom tercile family wealth (RR, 0.99; P = 0.002) was only significant for the Hui. In the multivariate model (Table 2, Panel B), parental out-migration (RR, 1.05; P = 0.031) was only significant for the Han, while bottom tercile standardized math score (RR, 0.95; P = 0.059) and no time spent watching television after school (RR, 1.06; P = 0.004) was only significant for the Hui.

In the simple regression model among children needing glasses (Table 3), ownership was positively associated with age (RR, 1.03; P = 0.006), female sex (RR, 1.17; P<0.001), parental education (RR, 1.20; P<0.001), parental out-migration (RR, 1.05; P = 0.034), no time spent watching television after school (RR, 1.12; P<0.001), spending at least one hour studying after school (RR, 1.294; P<0.001) and severe visual impairment (RR, 2.00; P<0.001). Glasses ownership was negatively correlated with thinking glasses worsen vision (RR, 0.81; P<0.001), believing eye exercises can correct myopia (RR, 0.82; P<0.001), bottom tercile standardized math score (RR, 0.74; P<0.001), bottom tercile family wealth (RR, 0.80; P<0.001), and being Hui (RR, 0.73; P<0.001).

**Table 3:** Simple model: Potential associations with self-reported glasses ownership among students with uncorrected visual acuity < = 6/12 in both eyes.

	Total			Han			Hui		
	RR	95% CI	P-value	RR	95% CI	P-value	RR	95% CI	P-value
Age, years	1.03	1.01,1.06	0.006	1.04	1.01,1.07	0.013	1.05	1.01,1.08	0.007
Female sex	1.17	1.11,1.24	<0.001	1.19	1.11,1.27	<0.001	1.22	1.13,1.31	<0.001
One or both parents with ≥9 years of education	1.20	1.13,1.28	<0.001	1.11	1.03,1.19	0.006	1.08	1.01,1.17	0.033
One or both parents out-migrated for work	1.05	1.00,1.11	0.034	1.05	0.99,1.11	0.129	1.05	0.98,1.12	0.211
Think glasses will harm vision	0.81	0.77,0.86	<0.001	0.81	0.75,0.87	<0.001	0.83	0.78,0.90	<0.001
Think eye exercises can correct myopia	0.82	0.78,0.87	<0.001	0.89	0.84,0.94	<0.001	0.79	0.73,0.85	<0.001
Standardized math score (bottom third)	0.74	0.69,0.79	<0.001	0.74	0.68,0.81	<0.001	0.77	0.71,0.84	<0.001
Family wealth (poorest third)	0.80	0.74,0.85	<0.001	0.86	0.79,0.93	<0.001	0.79	0.73,0.86	<0.001
No time spent watching television after school	1.12	1.07,1.18	<0.001	1.19	1.12,1.26	<0.001	1.04	0.98,1.11	<0.001
Time spent on studying after school >60 mins	1.29	1.22,1.37	<0.001	1.21	1.13,1.30	<0.001	1.27	1.17,1.37	<0.001
Severe vision impairment (≤6/60)	2.00	1.90,2.11	<0.001	1.76	1.68,1.85	<0.001	2.19	2.03,2.35	<0.001
Hui student	0.73	0.68,0.77	<0.001	-	-	-	-	-	-
	7494			3003			4491		

Table 4 reports the multivariate regression model results. Significant associations between glasses ownership and certain child characteristics persisted, such as age, female sex, parental out-migration, middle and top tercile math score compared to bottom, after school study, severe vision impairment, thinking glasses worsen vision, believing eye exercises can correct vision impairment, bottom tercile family wealth and being Hui (Table 4).

**Table 4:** Multivariate model: Potential associations with self-reported glasses ownership among students with uncorrected visual acuity < = 6/12 in both eyes.

	Total			Han			Hui		
	RR	95% CI	P-value	RR	95% CI	P-value	RR	95% CI	P-value
Age, years	1.06	1.04,1.08	<0.001	1.05	1.02,1.08	0.004	1.07	1.03,1.10	<0.001
Female sex	1.17	1.11,1.22	<0.001	1.14	1.07,1.21	<0.001	1.19	1.10,1.26	<0.001
One or both parents with ≥9 years of education	1.02	0.97,1.07	0.470	1.04	0.97,1.11	0.235	1.00	0.93,1.08	0.934
One or both parents out-migrated for work	1.05	1.01,1.10	0.022	1.05	0.99,1.12	0.074	1.05	0.98,1.12	0.146
Think glasses will harm vision	0.87	0.83,0.92	<0.001	0.86	0.80,0.92	<0.001	0.89	0.83,0.95	0.001
Think eye exercises can correct myopia	0.88	0.84,0.92	<0.001	0.94	0.89,1.00	0.038	0.83	0.77,0.89	<0.001
Standardized math score (bottom third)	Reference								
Middle third	1.17	1.10,1.25	<0.001	1.21	1.10,1.33	<0.001	1.15	1.05,1.25	0.003
Highest third	1.29	1.21,1.37	<0.001	1.28	1.17,1.41	<0.001	1.31	1.20,1.43	<0.001
Family wealth (poorest third)	0.85	0.80,0.89	<0.001	0.90	0.83,0.97	0.005	0.81	0.75,0.87	<0.001
Time spent on watching TV after school = none	1.04	1.00,1.09	0.071	1.09	1.03,1.16	0.002	1.00	0.94,1.07	0.960
Time spent on studying after school = none	Reference								
1–30 minutes	1.00	0.89,1.13	0.972	0.95	0.82,1.12	0.540	1.05	0.89,1.24	0.550
31–60 minutes	1.06	0.95,1.19	0.306	0.97	0.84,1.13	0.704	1.15	0.97,1.36	0.105
>60 minutes	1.16	1.04,1.30	0.009	1.07	0.93,1.23	0.359	1.26	1.06,1.50	0.009
Severe vision impairment (≤6/60)	1.90	1.80,2.01	<0.001	1.72	1.63,1.82	<0.001	2.11	1.97,2.25	<0.001
Hui student	0.79	0.74,0.83	<0.001	-	-	-	-	-	-
	7494			3003			4491		

## Discussion

The rates of visual impairment presented in this study (54.5% of Han and 45.2% of Hui children) are much higher than other LMICs around the world, while low relative to those in urban China. One study in Nepal using the same Refractive Error Studies in Children (RESC) protocol found that only 2.9% of children had visual acuity ≤6/12 [34]. Another RESC study in Malaysia found that 17.1% of school-aged children had uncorrected visual acuity ≤6/12 [35]. In urban China, visual impairment is much more common, as almost 75% of high school aged children suffer from myopia [17].

While the general glasses ownership rates found in this study are only slightly lower than among urban children [17], our findings agree with the literature that less attention has been paid to minority groups, as ownership rates for rural minorities in China are consistently low [36]. The glasses ownership rates found in our study are similar to comparable populations in other LMICs [35]. Given that the Hui population exceeds 10 million people [22], however, the number of Hui children without proper glasses is worrying. It is particularly concerning that our findings indicate glasses usage rates of Hui children in our sample are lower than Han children.

Even among the children who owned glasses in our sample, about 60% of them have glasses that did not optimally correct their vision. This rate of poor prescription is quite high, as other research report rates of poor prescriptions at 17.9% [37] and 26.9% [38]. There might be several reasons for this. One is that the vision of some children is simply not correctable by glasses. While this is true, prior studies suggest this proportion is around only 1% [17, 32]. Alternatively, rural refractionists may not be providing correct prescriptions to the children in our sample. This is more likely, as the competence of rural refractionists has been shown to be less than that of their urban counterparts [37, 39]. A third possibility is that children may have simply outgrown their prescriptions due to myopic progression, and have not received new ones, as children in rural China tend to outgrow their prescriptions in about one year [37]. It is likely that all three of these factors are at play here.

The correlations we find in this study between individual characteristics and visual impairment are largely expected and agree with the literature. From the standpoint of implementing myopia control programs such as the current Chinese national effort, the inverse association of myopia with time spent outdoors [40] and the positive association with time spent reading [41, 42] are of particular importance. An inverse relationship between watching television and prevalence of myopia agrees with these results as well, as studying for school may be replacing the watching of television, increasing time spent on near-work. Measures to reduce early demand for reading by limiting homework for young children, and to increase time outdoors to two hours per day are integral to the current Chinese national program [19]. The correlation between sex and visual impairment is also not surprising, as girls, especially in China, spent more time studying [43].

The associations with glasses usage presented in this study reveal the barriers to eye care apparent in rural contexts: poverty, poor education, and incorrect beliefs about vision care, namely believing that wearing eyeglasses worsens myopia and that eye exercises can improve myopia. These barriers are more prevalent among the Hui than the Han. These findings are consistent with prior research showing that barriers to glasses usage include price, parental disagreement, and fear of weakening vision [43]. Additionally, that sex and severe visual impairment are correlated with glasses usage comes as no surprise, as simply having visual impairment creates the need for the usage of eyeglasses. As demonstrated above, female students in this sample were more likely to be visually impaired.

This study contributes to the literature by examining visual impairment and glasses usage in a large, population-based sample of junior high school students, primarily made up of an understudied ethnic minority. The shortcomings of this study include its cross-sectional design and its exclusive focus on the Ningxia Hui Autonomous Region. This exclusive focus may restrict the generalizability of our results to other minority populations in rural China.

Many barriers to vision care are present in China. Large steps have been taken towards combatting this problem, like the comprehensive national plan that was launched in 2018 designed to reduce and control myopia in children [44]. This plan introduces concrete steps aimed at myopia control, including reductions in written homework and increased daily time outdoors, with a national goal of reducing the prevalence of myopia by 0.5% per year between 2018 and 2023 [19]. However, our study shows that many barriers are still present in rural areas. To efficiently overcome these barriers, policymakers should focus on improving vision care in rural areas, especially for ethnic minorities. In particular, future glasses interventions and policy designed to improve glasses usage should focus on populations with lower incomes and seek to correct erroneous beliefs about the safety of glasses and efficacy of traditional eye exercises. These recommendations are particularly relevant for policy focused on ethnic minorities like the Hui.
